# Systematic review of therapeutic nipple‐sparing *versus* skin‐sparing mastectomy

**DOI:** 10.1002/bjs5.50119

**Published:** 2018-12-19

**Authors:** R. A. Agha, Y. Al Omran, G. Wellstead, H. Sagoo, I. Barai, S. Rajmohan, M. R Borrelli, M. Vella‐Baldacchino, D. P. Orgill, J. E. Rusby

**Affiliations:** ^1^ Department of Plastic Surgery, Royal Free NHS Foundation Trust London UK; ^2^ GKT School of Medical Education, King's College London London UK; ^3^ Imperial College School of Medicine London UK; ^4^ Department of Plastic Surgery University Hospitals of North Midlands Stoke‐on‐Trent UK; ^5^ Ear, Nose and Throat Department, Norfolk and Norwich University Hospitals NHS Foundation Trust Norwich UK; ^6^ Department of Orthopaedics, Oxford University Hospitals NHS Foundation Trust Oxford UK; ^7^ Breast Surgical Unit, The Royal Marsden NHS Foundation Trust Sutton UK; ^8^ Division of Plastic Surgery, Stanford University Stanford California USA; ^9^ Division of Plastic Surgery, Brigham and Women's Hospital Boston Massachusetts USA

## Abstract

**Background:**

The use of nipple‐sparing mastectomy (NSM) is increasing, despite unproven oncological safety in the therapeutic setting. The aim of this systematic review was to determine the safety and efficacy of NSM compared with skin‐sparing mastectomy (SSM).

**Methods:**

A literature search of all original studies including RCTs, cohort studies and case–control studies comparing women undergoing therapeutic NSM or SSM for breast cancer was undertaken. Primary outcomes were oncological outcomes; secondary outcomes were clinical, aesthetic, patient‐reported and quality‐of‐life outcomes. Data analysis was undertaken to explore the relationship between NSM and SSM, and preselected outcomes. Heterogeneity was assessed using the Cochrane tests.

**Results:**

A total of 690 articles were identified, of which 14 were included. There was no statistically significant difference in 5‐year disease‐free survival and mortality for NSM and SSM groups, where data were available. Local recurrence rates were also similar for NSM and SSM (3·9 *versus* 3·3 per cent respectively; *P* = 0·45). NSM had a partial or complete nipple necrosis rate of 15·0 per cent, and a higher complication rate than SSM (22·6 *versus* 14·0 per cent respectively). The higher overall complication rate was due to the rate of nipple necrosis in the NSM group (15·0 per cent).

**Conclusion:**

In carefully selected cases, NSM is a viable choice for women with breast cancer who need to have a mastectomy. More research is needed to help further refine which surgical approaches to NSM optimize outcomes.

## Introduction

Breast cancer is the most common cancer in the UK, accounting for 31 per cent of all new cancer cases and a lifetime incidence of one in eight in women^1^. Approximately 50 000 women are diagnosed with breast cancer each year, 16 000 of whom will undergo a mastectomy. Every year, breast cancer is associated with nearly 12 000 deaths^2^. According to the National Cancer Institute^3^, there were 232 340 new cases and 39 620 deaths from breast cancer in 2013 in the USA, with more than 96 000 women undergoing breast reconstruction following surgery.

From its inception in 1894 until the 1960s, Halsted's radical mastectomy was the standard of care for patients. Later, the modified radical mastectomy (MRM) was described by Patey; this achieved a local recurrence rate of 10 per cent after 10 years^4^. In 1991, Toth and Lappert^5^ described skin‐sparing mastectomy (SSM); this procedure involves removing the entire breast and nipple–areola complex (NAC) while maintaining the skin envelope and the native inframammary crease (IMC). A meta‐analysis^6^ in 2010 found that local recurrence rates after SSM were equivalent to those after MRM. This analysis had several limitations, however, including different follow‐up times for SSM and MRM, and the groups were not matched for prognostic factors.

The principal reason why a surgeon may want to preserve a nipple is aesthetic, with studies reporting improved patient satisfaction and psychological benefit^7^. The nipple is one of the crucial defining visual features of a breast^8^. With removal of the NAC, there is a loss of the point in the profile of the breast at which the most natural convexity occurs. Preserving the NAC also removes the need for staged nipple reconstruction and areola tattooing, after which there can be loss of projection and fading over time, respectively.

Historically, mastectomy has included resection of both the NAC and the gland, the concern being that the NAC may harbour occult tumour cells. Indeed, large trials have reported the NAC to be involved in 5–12 per cent of cases^9^, with some reports giving rates as high as 58 per cent^10^. The initial report of nipple‐sparing mastectomy (NSM) came in 1984 from Hinton and colleagues^11^, who found that NSM could achieve comparable local recurrence and survival rates to those for MRM. Despite this, the technique did not attain widespread use at the time owing to oncological concerns, which persist today^10^ ^12^. Similar concerns were raised over the oncological safety of breast‐conserving surgery for small tumours until Veronesi and colleagues' seminal RCTs were published^13^, which showed equivalent oncological outcomes for breast‐conserving surgery and mastectomy for small tumours, now with 20‐year follow‐up. Treatments for breast cancer have become more nuanced over the past few decades, and are tailored to individuals with care directed through multidisciplinary teams.

Previous systematic reviews^14^, ^15^, ^16^ have assessed the oncological safety of therapeutic NSM (*Table* *S1*, supporting information). Their quality is assessed in *Table S2* (supporting information) using the validated assessment tool Assessment of Multiple Systematic Reviews (AMSTAR)^17^, ^18^, ^19^. In summary, these previous systematic reviews were not fully AMSTAR‐compliant.

Traditionally, progression to less radical or invasive surgical management of breast cancer has not produced worse outcomes, although this has coincided with extension of the use of systemic treatment and irradiation. Lanitis and colleagues' meta‐analysis^20^ comparing SSM with conventional mastectomy showed no significant difference in local recurrence rates. However, concerns still exist that occult malignancy could remain within residual glandular breast tissue. Despite these concerns, the US National Cancer Institute Surveillance, Epidemiology, and End Results (SEER) database demonstrated a 202 per cent increase in NSM procedures between 2005 and 2009^21^.

NSM is an active research front. A basic search using the database Scopus for ‘nipple‐sparing mastectomy’ reveals how research and interest in this area has increased over the past 21 years, from no indexed articles in 1995 to more than 80 in 2016 (*Fig. S1*, supporting information).

Since the most recent systematic review of NSM completed its search in January 2014, more than 100 articles have been published in this area^14^. A new systematic review that is AMSTAR‐compliant is thus warranted to update the understanding of this rapidly changing field and potentially answer questions that previous studies failed to do. The aim of this study was to perform a systematic review of the safety and efficacy of therapeutic NSM with a particular focus on oncological, clinical, aesthetic, patient‐reported and quality‐of‐life outcomes.

## Methods

This review was conducted in line with the recommendations specified in the Cochrane Handbook for intervention reviews version 5.1.0^22^ and is AMSTAR‐compliant. It is reported in line with the Preferred Reporting Items for Systematic Reviews and Meta‐Analyses (PRISMA) statement^23^. A protocol was registered on the Research Registry UIN (reviewregistry29; https://www.researchregistry.com) and published *a priori* 24.

### Studies and participants

All comparative studies with levels of evidence 1–3, as defined by the Oxford Centre for Evidence‐Based Medicine^25^ (RCTs, cohort and case–control studies), were included, and single‐group cohorts, case series, case reports and expert opinions were excluded. Only articles that mentioned one or more of the outcomes of interest were included. Duplicate studies were excluded, as were cost‐effectiveness studies not reporting original data and purely technical descriptions.

Women undergoing mastectomy for breast cancer were included. Men and transgender patients were excluded.

### Interventions and comparators

The intervention of interest was NSM, which involves removal of all glandular breast tissue with preservation of the native skin envelope, IMC and nipple. The comparator was SSM, which involves the removal of all glandular breast tissue and the nipple, with preservation of the native skin envelope and IMC.

### Outcome measures

Primary outcomes were overall survival and local recurrence rate in the follow‐up interval. Secondary outcomes were clinical complications (such as NAC or skin flap necrosis), haematoma, seroma, infection and readmission to hospital. Additional outcomes included aesthetic outcome as judged by the instrument used in the study, patient‐reported outcomes (such as patient satisfaction), and quality‐of‐life outcomes (for example, psychological well‐being, impact on body image, relationships and sexuality, using instruments such as EQ‐5D™ (EuroQol Group, Rotterdam, the Netherlands)).

### Search methods

The following electronic databases were searched from inception to end of June 2017: MEDLINE; Embase; SciELO (Scientific Electronic Library Online); the Cochrane Library, including the Cochrane Database of Systematic Reviews, Cochrane Central Register of Controlled Trials, DARE (Database of Abstracts of Reviews of Effect) and Cochrane Methodology Register; Health Technology Assessment Database; National Health Service Economic Evaluation Databases and Cochrane Groups; ClinicalTrials.gov; and the WHO International Clinical Trials Registry Platform.

The search was conducted by an information specialist experienced in systematic review, using appropriate keywords in the English language combined with Boolean logical operators. The search strategy for MEDLINE is shown in *Table S3* (supporting information).

Searches were translated to the appropriate syntax of other databases, using free text and the relevant database thesaurus terms. The search was not limited by language.

Grey literature searches included a search of Open Grey (http://www.opengrey.eu). In addition, references of all included papers and previous systematic reviews were searched for relevant studies.

Studies identified through the electronic and manual searches were listed within a Microsoft Excel® 2011 database (Microsoft, Redmond, Washington, USA). Duplicate articles were excluded. Articles were selected for inclusion via two steps. Titles and abstracts were screened by two researchers, and discrepancies were resolved through discussion; where agreement could not be reached, the article proceeded to the next stage. Next, the full texts of selected articles were downloaded and further assessed for inclusion by two researchers. When discrepancies could not be resolved through consensus, a senior author made the final decision on inclusion.

### Data extraction, collection and management

Data extraction was performed independently by two teams of researchers. Each team screened all articles on the database. The work was split equally and distributed within each team. Any discrepancies that arose between teams proceeded to full‐text screening stage. If discrepancies persisted, they were resolved by consensus. Where required, data were reviewed by two senior authors. Data were extracted into a standardized Microsoft Excel® 2011 database. The data extraction template was first piloted by extracting a smaller sample of papers. This not only provided training to the extractors but also helped to ensure uniformity of extraction and identify issues early on, for example how to document aesthetic outcomes and quality of life. Authors were contacted to ascertain individual values for NSM and SSM where possible.

### Assessment of risk of bias

Non‐randomized studies were scored according to the Cochrane risk‐of‐bias assessment tool ACROBAT‐NRSI^26^. Study protocols were compared with final papers where possible. Relevant missing information across all study types is presented.

Funnel plots were generated to determine whether there was a skew towards positive outcomes, thereby assessing for publication bias^27^.

### Statistical analysis

NSM and SSM were compared. Using Review Manager 5.2.6 (RevMan)^28^, an assessment of heterogeneity in comparative studies was made. If high, as defined by the *I* ^2^ statistic (*I* ^2^ above 50 per cent), meta‐analysis was performed using a random‐effects model; otherwise a fixed‐effect model was used^29^.

The intention was to perform an additional analysis to assess whether particular oncological profiles are associated with better outcome in NSM; however, this was not possible due to insufficient detail.

## Results

**Figure 1:**
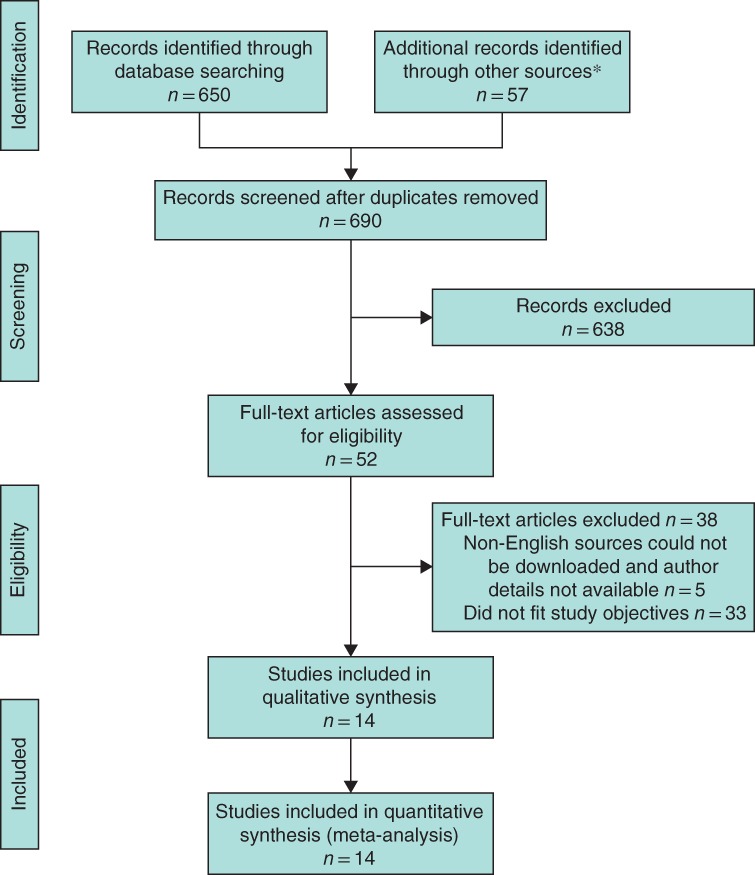
PRISMA flow chart showing selection of articles for review. *Two records identified by hand‐searching, 35 from previous systematic reviews and 20 registered trials

A total of 690 articles were identified, of which 14 were included (*Fig*. 1). These 14 studies^30^, ^31^, ^32^, ^33^, ^34^, ^35^, ^36^, ^37^, ^38^, ^39^, ^40^, ^41^, ^42^, ^43^ included 3015 breasts, of which there were 1419 NSMs and 1596 SSMs; follow‐up ranged from 18 to 101 months.

All studies represented level 3 evidence and, apart from one case–control study, were retrospective cohort studies.

*Table* 1 shows the Cochrane risk‐of‐bias tool results for the included studies; a summary of the included papers is given in *Table S4* (supporting information). Oncological profiles for the included studies can be found in *Table S5* (supporting information). The mortality and local recurrence outcomes are shown in *Table* 2, and the nipple necrosis and complications for NSM and SSM groups in *Table* 3. *Table S6* (supporting information) summarizes complications across the NSM and SSM groups, and aesthetic and patient‐reported outcomes are shown in *Table S7* (supporting information).

**Table 1:** Methodological bias of included studies using the Cochrane risk‐of‐bias tool for non‐randomized studies

Reference	Confounding	Participant selection	Measure bias	Departure from intentions	Missing data	Measurement of outcomes	Selection of result	Overall bias
Wei *et al*.^30^	Moderate	Moderate	Low	Low	Low	Low	Moderate	Moderate
Lemaine *et al*.^31^	Moderate	Moderate	Moderate	Low	Moderate	Serious	Moderate	Moderate
Kim *et al*.^32^	Moderate	Serious	Low	Moderate	Low	Low	Moderate	Serious
Poruk *et al*.^33^	Moderate	Moderate	Low	Low	Low	Low	Moderate	Moderate
Yoo *et al*.^34^	Moderate	Moderate	Low	Moderate	Moderate	Low	Low	Moderate
Gould *et al*.^35^	Low	Low	Low	Low	Low	Low	Moderate	Low
Burdge *et al*.^36^	Moderate	Low	Low	Low	Low	Serious	Low	Serious
Moyer *et al*.^37^	Moderate	Low	Low	Moderate	Low	Moderate	Low	Moderate
Boneti *et al*.^38^	Moderate	Moderate	Low	Low	Low	Low	Moderate	Moderate
Jeon *et al*.^39^	Moderate	Moderate	Moderate	Moderate	Low	Low	Moderate	Moderate
Kim *et al*.^40^	Low	Moderate	Moderate	Moderate	Moderate	Low	Low	Moderate
Gerber *et al*.^41^	Low	Serious	Low	Moderate	Low	Low	Moderate	Moderate
Ueda *et al*.^42^	Moderate	Serious	Low	Low	Low	Moderate	Moderate	Moderate
Gerber *et al*.^43^	Low	Serious	Low	Moderate	Low	Low	Moderate	Moderate

**Table 2:** Mortality and local recurrence outcomes

	Duration of follow‐up (months)*		Mortality (%)		Local recurrence (%)	
Reference	NSM	SSM	NSM	SSM	NSM	SSM
Wei *et al*.^30^	18 (5–83)	33 (5–84)	n.r.	n.r.	n.r.	n.r.
Lemaine *et al*.^31^	n.r.	n.r.	n.r.	n.r.	n.r.	n.r.
Kim *et al*.^32^	42 (24–61)		n.r.	n.r.	n.r.	n.r.
Poruk *et al*.^33^	26(18)†	30(16)†	3·1 (4 of 130)	16·0 (21 of 131)	1·5 (2 of 130)	8·4 (11 of 131)
Yoo *et al*.^34^	31† (7–84)		n.r.	n.r.	1·0 (4 of 383)	2·1 (12 of 581)
Gould *et al*.^35^	n.r.	n.r.	n.r.	n.r.	n.r.	n.r.
Burdge *et al*.^36^	25(19)†	38(26)†	2 (1 of 60)		10 (4 of 39)‡	14 (3 of 21)
Moyer *et al*.^37^	≥ 6		n.r.	n.r.	n.r.	n.r.
Boneti *et al*.^38^	25 (3–102)	38 (4–144)	n.r.	n.r.	4·6 (7 of 152)§	5·0 (7 of 141)
Jeon *et al*.^39^	71†	60†	7·9	4·8	9·0 (12 of 133)	6 (4 of 69)
Kim *et al*.^40^	60	67	2·9¶	2·7¶	2·0 (3 of 152)	0·8 (3 of 368)
					1·3 (2 of 152) in nipple	
Gerber *et al*.^41^	101† (26–156)		22 (13 of 60)	21 (10 of 48)	12 (7 of 60)	10 (5 of 48)
Ueda *et al*.^42^	53†	47†	n.r.	n.r.	9 (3 of 33)	2 (1 of 41)

* Values are median (range) unless indicated otherwise;

† values are mean(s.d.).

‡ No recurrences or occurrences in the spared nipple–areola complex or the scar of the nipple‐sparing mastectomy (NSM).

§ None in the nipple.

¶ Five‐year estimates provided for mortality. SSM, skin‐sparing mastectomy; n.r., not reported.

**Table 3:** Nipple necrosis and complications for nipple‐sparing and skin‐sparing mastectomy groups

	NSM		SSM	
Reference	Complication	No.	Complication	No.
Wei *et al*.^30^	Partial nipple necrosis	2 of 52 (4)	Full‐thickness mastectomy flap necrosis	12 of 202 (5·9)
	Full‐thickness mastectomy flap necrosis	4 of 52 (8)	Seroma	4 of 202 (2·0)
	Haematoma requiring return to operating theatre	1 of 52 (2)	Haematoma requiring return to theatre	4 of 202 (2·0)
	Infection requiring antibiotics	1 of 52 (2)	Infections requiring antibiotics	2 of 202 (1·0)
	None	43 of 52 (83)	None	180 of 202 (89·1)
Lemaine *et al*.^31^	Partial/full nipple necrosis	42 of 72 (58)	Partial/full‐thickness mastectomy skin flap necrosis	15 of 103 (14·6)
	Partial/full‐thickness mastectomy skin flap necrosis	8 of 72 (11)		
Kim *et al*.^32^	n.r.	n.r.	n.r.	n.r.
Poruk *et al*.^33^	n.r.	n.r.	n.r.	n.r.
Yoo *et al*.^34^	n.r.	n.r.	n.r.	n.r.
Gould *et al*.^35^	Total nipple necrosis	2 of 113 (1·8)	Overall complication rate (no necrosis)	32 of 120 (26·7)
	Partial nipple necrosis	21 of 113 (18·6)		
	Overall complication rate	32 of 113 (28·3)		
Burdge *et al*.^36^	Overall complication rate (no nipple necrosis reported)	12 of 39 (31)	Overall complication rate	8 of 21 (38)
Moyer *et al*.^37^	Partial nipple necrosis	15 of 40 breasts (38)	n.r.	
	Skin flap necrosis	1 of 40 (3)		
	Major complications necessitating reoperation	3 of 26 patients (12)		
	Latissimus flap to salvage exposed expander	1 of 26 (4)		
Boneti *et al*.^38^	Nipple necrosis	2 of 281 (0·7)	Skin flap ischaemia	7 of 227 (3·1)
	Implant removal	2 of 281 (0·7)	Postoperative infection	6 of 227 (2·6)
	Skin flap ischaemia	13 of 281 (4·6)	Implant removal	3 of 227 (1·3)
	Postoperative complication	5 of 281 (1·8), infection	Haematoma requiring re‐exploration	1 of 227(0·4)
	Overall complication rate	20 of 281 (7·1)	Overall complication rate (no nipple necrosis)	14 of 227 (6·2)
	Postoperative bleeding requiring re‐exploration	2 of 281 (0·7)	Overall complication via elliptical incision	9 of 193 (4·7)
	Overall complication via infra‐areolar incision	9 of 139 (6·5)	Overall complication via inverted T incision	1 of 16 (6)
	Overall complication via inframammary incision	9 of 105 (8·6)	Overall complication via lollipop incision	4 of 16 (25)
	Overall complication via previous scar	1of 23 (4)	Overall complication via inverted T incision	1 of 16 (6)
	Overall complication via axillary incision	1 of 12 (8)	Overall complication via lollipop incision	4 of 16 (25)
Jeon *et al*.^39^	n.r.	n.r.	n.r.	n.r.
Kim *et al*.^40^	Total nipple necrosis	11 of 115 (9·6)	n.r.	n.r.
	Partial nipple necrosis	15 of 115 (13·0)		
Gerber *et al*.^41^	n.r.	n.r.	n.r.	
Ueda *et al*.^42^	n.r.	n.r.	n.r.	
Gerber *et al*.^43^	Nipple necrosis	6 of 61 (10)	Infection	3 of 51 (6)
	Infection	5 of 61 (8)	Thrombosis/emboli	2 of 51 (4)
	Thrombosis/emboli	3 of 61 (5)	Haematoma/bleeding during operation	2 of 51 (4)
	Hematoma/bleeding during operation	1 of 61 (2)	Blood transfusion	3 of 51 (6)
	Blood transfusion	2 of 61 (3)		
Total	Partial or complete nipple necrosis rate	116 of 773 (15·0)		
	Skin flap necrosis rate	26 of 773 (3·4)	Skin flap necrosis rate	28 of 724 (3·9)
	Overall complication rate	175 of 773 (22·6)	Overall complication rate	101 of 724 (14·0)

Results are reported as number of patients or breasts, as indicated. Values in parentheses are percentages. NSM, nipple‐sparing mastectomy; SSM, skin‐sparing mastectomy; n.r., not reported.

**Figure 2:**
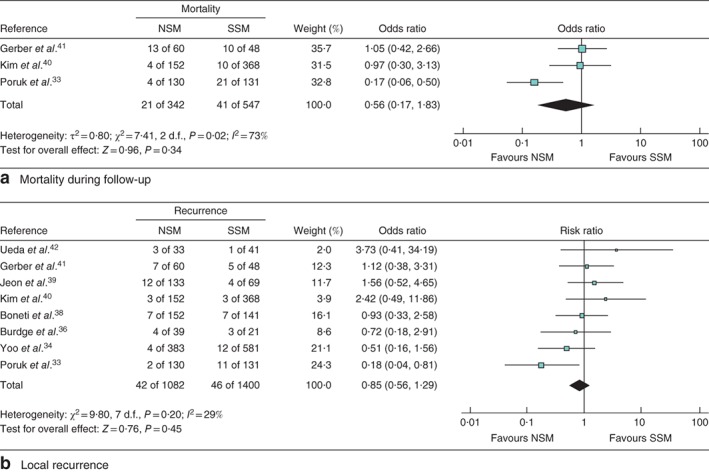
Forest plots comparing outcomes for nipple‐sparing *versus* skin‐sparing mastectomy. **a** Mortality in the follow‐up interval and **b** local recurrence outcomes for nipple‐sparing mastectomy (NSM) *versus* skin‐sparing mastectomy (SSM). Mantel–Haenzsel random‐effects (**a**) and fixed‐effect (**b**) models were used. Odds ratios and risk ratios are shown with 95 per cent confidence intervals

*Fig*. 2 *a* is a forest plot of mortality for NSM *versus* SSM; there was no statistically significant difference (*P* = 0·34). A random‐effects analysis was performed due to significant heterogeneity. Local recurrence for NSM *versus* SSM in shown in *Fig*. 2 *b*; again, there was no significant difference (*P* = 0·45). A fixed‐effect model was used as the *I* ^2^ statistic was 29 per cent.

## Discussion

Fourteen studies including 3015 breasts were included in this systematic review. There were no significant differences between NSM and SSM groups in local recurrence rate (3·9 and 3·3 per cent respectively; *P* = 0·45) or mortality outcomes (*P* = 0·34). The results of a systematic review are inevitably dependent on the quality of the studies included. In this context, it is possible that a difference does exist, but is obscured by selection bias and differences in duration of follow‐up. Nonetheless, it is unlikely to be large or clinically significant. NSM does have a higher complication rate of 22·6 per cent, compared with 14·0 per cent for SSM, largely due to a partial or complete nipple necrosis rate of 15·0 per cent, which is, by definition, avoided by sacrificing the nipple.

There is, however, a lower rate of mastectomy skin flap necrosis (MSFN) for NSM (3·4 per cent *versus* 4·7 per cent for SSM). Unfortunately, this complication was sparsely reported, and this result is driven by the study of Lemaine and colleagues^31^, who reported a 67 per cent rate of MSFN and had specifically set out to classify the extent and severity of this complication. In most other studies, data were not available on the severity or consequences of MSFN, for example whether it required surgical debridement or led to loss of an underlying implant. Given the wide range of incidence (0–67 per cent), it is likely that these studies are reporting differently.

NSM had better aesthetic outcomes than SSM in the six studies^30^ ^36^, ^37^, ^38^ ^41^, ^43^ that assessed this. In addition, Wei and colleagues^30^ used the BREAST‐Q and reported better psychological and sexual scores for NSM compared with SSM, although the difference in duration of follow‐up may have confounded this finding. Some studies, such as that of Boneti *et al*.^38^, showed low rates of data availability for patient‐reported satisfaction (30 per cent for NSM and 8 per cent for SSM), increasing the risk of bias.

A recently published Cochrane review^44^ assessed NSM, SSM and also traditional MRM. It included 11 cohort studies; searches were concluded on 30 September 2014, nearly 3 years before the present review, hence the inclusion of fewer studies. The Cochrane review results were inconclusive for differences between rates of local recurrence and adverse events for the three types of mastectomy. Owing to a lack of numerical data it was not possible to pool the results of the different studies. The conclusion^44^ was: ‘In practice, the decision to select NSM over other types of mastectomy should be done through shared decision‐making after extensive discussion of the risks and benefits. Generally, the NSM studies reported a favourable aesthetic result and a gain in quality of life compared with the other types of mastectomy.’

Headon and co‐workers^45^ conducted a systematic review of NSM with a pooled analysis of 12 358 procedures. The overall pooled locoregional recurrence rate was 2·4 per cent (compared with 3·9 per cent in the present review), the overall complication rate was 22·3 per cent (compared with 22·6 per cent) and partial or total nipple necrosis was 5·9 per cent (compared with 15·0 per cent in the present review). The recurrence rate was lower than that in the present review, but the range of follow‐up was lower at 7·4–156 months, which may provide an explanation. The conclusion^45^ was: ‘NSM appears to be an oncologically safe option for appropriately selected patients, with low rates of locoregional recurrence. For NSM to be performed, tumours should be peripherally located, smaller than 5 cm in diameter, located more than 2 cm away from the nipple margin, and human epidermal growth factor 2‐negative. A separate histopathological examination of the subareolar tissue and exclusion of malignancy at this site is essential for safe oncological practice.’

These findings support the role of NSM in modern breast surgery practice for selected patients. *Table S6* (supporting information), which summarizes complication rates for NSM and SSM, provides useful data for discussion when counselling women for these two techniques as part of the informed consent process. Smokers and diabetic patients, as well as women with large ptotic breasts or those who have undergone radiotherapy, are most likely to have complications such as nipple necrosis or skin flap necrosis with NSM, although the data are not strong or homogeneous enough to show statistically robust correlations. Interestingly, albeit in patients without cancer, residual NAC sensitivity after NSM was low compared with that in a non‐operated control group; thus, patients may also need to be counselled for this^46^. Where the woman's desire to maintain a nipple needs to be balanced against blood supply and oncological concerns, clinicians may consider doing a ‘delay procedure’, which allows for a retroareolar biopsy at the first stage before deciding whether to proceed with NSM or SSM after discussion with the patient, a position advocated by Karian and co‐workers^47^. Given that false‐negative rates for frozen section have been reported to be as high as 15·4 per cent, this may be prudent in such patients^48^. In addition, intraoperative irradiation, the delivery of a single dose of radiation to the periphery of the tumour bed in the immediate intraoperative time frame, may also be considered. Two large prospective randomized trials^49^ ^50^ have established the safety and efficacy of breast intraoperative irradiation in early‐stage breast cancer; this technique has the advantage of preserving breast tissue, mitigating toxicity and reducing the inconvenience of lengthier radiotherapy treatment courses, and so may be prudent in NSM^54^.

Given the limitations of reporting of complications for NSM, the imminent publication of the UK prospective audit on implant‐based breast reconstruction (the i‐BRA study) may provide more reliable data for NSM and SSM. Further research is needed to help refine the evidence on surgical approaches for NSM to optimize outcomes. For example, knowledge of which incisions minimize nipple and mastectomy skin flap necrosis is needed. Greater understanding of the NAC blood supply and how the various incisions can disrupt this, the interplay of reconstructive techniques with perfusion of skin, and other methods to optimize skin flap viability is required, as well as methods to increase its perfusion. There are no data on learning curves for performing NSM.

Gould and colleagues' work^35^, showing no impact of surgical technique and biomaterials used for reconstruction on rates of nipple necrosis, is welcome, but the sample size was small and more data are needed to achieve significant power, minimize the impact of confounders and determine statistical significance. The traditional role of an acellular dermal matrix (ADM) in defining the lower implant pocket and offloading the lower pole may help to reduce the pressure an implant applies to the NAC and mastectomy skin envelope, thereby increasing blood supply and reducing rates of necrosis^51^. This role of ADMs in NSM has not been fully elucidated. Given the heterogeneity in the instruments used to determine aesthetic and patient satisfaction, further research using standardized instruments, such as BREAST‐Q, that allow comparison is welcome.

This is the most comprehensive systematic review of NSM *versus* SSM to date. A rigorous search was performed for evidence to the end of June 2017, using two independent search teams. A protocol for this study was published *a priori*; it followed Cochrane methodology and is reported in line with PRISMA criteria. This review was not restricted to the English language, and included 3015 breasts from North America, Japan, South Korea and Germany. It scored 11 of 11 on AMSTAR criteria^18^.

There were no RCTs suitable for inclusion in the review, and no prospective studies. All included studies were of evidence level 3, and their quality was variable. Using the Cochrane ACROBAT‐NRSI tool, all articles except one^35^ were judged to have a moderate or serious risk of bias. Systematic review authors cannot include studies that they are unable to access; thus libraries and the authors themselves were contacted in an effort to maximize inclusivity. When asked for clarifications, study authors rarely responded, hampering efforts to analyse the data more deeply.

Many of the studies did not provide complete, clear or transparent information. These issues have been highlighted in previous systematic reviews and studies of reporting quality^52^, ^53^, ^54^. Average follow‐up ranged from 18 to 101 months, and was often not reported separately for NSM and SSM cohorts; thus, assessing differences in local or distant recurrence was impossible for some studies, and meaningless for studies at the shorter end of the follow‐up range. Furthermore, satisfaction can often decrease with time (as seen for Gerber and colleagues, 2003 and 2009^41^ ^43^).

Case mix and selection bias are important. The NSM group included more prophylactic cases, which are likely to have a lower local recurrence rate than patients with cancer, but significant limitations in the data prevented a sensitivity analysis to look at cancer cases alone. Even within the cancer cohorts, different stages of disease and durations of follow‐up challenge the analysis in a systematic review. The differences highlighted could certainly impact on the results. The Gerber study of 2003^41^ had significant overlap in patient population with the 2009 study^43^, so oncological outcomes were included from 2009 and not 2003, although complications were reported in 2003, and not in 2009.

The reporting of complications is very variable, with some studies reporting by patient and others by breast. Nonetheless, as these studies reported in the same way for SSM as NSM, they were still included in the analysis of complications. Finally, there is concern about the exclusion of 93 patients in the study by Lemaine *et al*.^31^ because they did not have clinical photographs within 90 days of surgery. Again, this may represent selection bias as patients with MSFN are more likely to have had photographs to document the complication.

Most studies did not report on aesthetic outcome. Those that did used a variety of assessment tools, making comparisons difficult. The lack of reporting of a minimum set of outcomes in a standard way often hampers systematic reviews. It is anticipated that publication bias might be a factor, given all studies should be reporting survival and only five^33^ ^36^, ^39^, ^40^, ^41^ did. Recently developed reporting guidelines, such as PROCESS (Preferred Reporting Case Series in Surgery) and STROCSS (Strengthening the Reporting of Cohort Studies in Surgery), are recommended to encourage more complete and transparent reporting^55^ ^56^. In addition, core outcome sets should be developed and used whenever possible^57^, and progress has been made in the field of breast reconstruction with the BRAVO (Breast Reconstruction And Valid Outcomes) core outcome set^58^.

This review provides an up‐to‐date summary of outcomes for NSM relative to the current standard of SSM. NSM is an option for selected women with breast cancer requiring mastectomy: those with no clinical or radiological evidence of nipple involvement. Women should be counselled about the risk of occult involvement, possibly with personalized risk assessment^59^. Women should be advised that there may be a recommendation for later removal of the nipple if it is found to be involved, but that systematic reviews such as this one have shown similar local recurrence, 5‐year disease‐free survival and mortality outcomes for NSM compared with SSM. This review will also help clinicians explain the higher complication rate in NSM, which is due to nipple necrosis, whereas rates of other complications are comparable, and there is evidence of better aesthetic outcomes. Risk factors leading to a poor outcome must be studied further so that patients can be advised as individuals. As with any oncological procedure, a high‐quality consent process, robust multidisciplinary team discussion and shared decision‐making are all important. All women consenting to NSM should be informed of the alternatives of skin‐sparing or simple mastectomy before making their decision. Further evidence on patient selection and surgical approaches to NSM will optimize outcomes and minimize complication rates.

## Supporting information


**Table S1** Previous reviews of nipple‐sparing mastectomy
**Table S2** Quality of previous systematic reviews according to AMSTAR
**Table S3** Electronic search strategy
**Table S4** Characteristics of the included studies
**Table S5** Oncological profiles of patients included in the studies
**Table S6** Summary of complication rates in NSM and SSM groups (breasts)
**Table S7** Clinician and patient‐reported outcomes
**Fig. S1** Number of articles published per year and indexed by Scopus under the search term ‘nipple‐sparing mastectomy’
