# Supplementary material for: Systematic review of therapeutic nipple‐sparing versus skin‐sparing mastectomy
Source: BJS Open. 2018 Dec 19;3(2):135–45. doi: 10.1002/bjs5.50119 (PMC6433323; doi:10.1002/bjs5.50119)
Supplement: Supplementary file 1 — Table S1 Previous reviews of nipple‐sparing mastectomy Table S2 Quality of previous systematic reviews according to AMSTAR Table S3 Electronic search strategy Table S4 Characteristics of the included studies Table S5 Oncological profiles of patients included in the studies Table S6 Summary of complication rates in NSM and SSM groups (breasts) Table S7 Clinician and patient‐reported outcomes Fig. S1 Number of articles published per year and indexed by Scopus under the search term ‘nipple‐sparing mastectomy’ [file BJS5-3-135-s001.docx]

**BJS5_50119**

**Systematic review of therapeutic nipple-sparing *versus* skin-sparing mastectomy**

**R. A. Agha, Y. Al Omran, G. Wellstead, H. Sagoo, I. Barai, S. Rajmohan, M. R Borrelli, M. Vella-Baldacchino, D. P. Orgill and J. E. Rusby**

**Table S1 Previous reviews of nipple-sparing mastectomy**

| REVIEW | DATABASES INCLUDED AND YEARS SEARCHED | STUDIES/ PATIENTS INCLUDED | KEY FINDINGS |
| --- | --- | --- | --- |
| Zhang 2015^14^ | **PubMed, 1978-2014** | **27 studies, 7971 patients** | “Due to the statistical heterogeneity detected with certain parameters, further investigations to confirm their association with nipple involvement [NI] will be needed.  Patients with one or more risk factors such as centrally located tumours; higher tumour stage; large tumours; ER negative/PR negative/HER positive status and associated DCIS have higher risk of NI.  Taking these factors into consideration comprehensively may help with decision-making process for NSM.” |
| Piper 2013^15^ | **MEDLINE and Cochrane databases, inception to Nov 2011** | **27 studies, 3331 mastectomies** | “Review of oncologic outcomes in the 10 studies [representing 1148 mastectomies] with documented mean/median follow-up of 2 years demonstrated an overall loco-regional recurrence rate of 2.8%.  Ischaemic complications involving the NAC were reported in 24 studies [representing 3091 mastectomies], with 9.1% of cases reported to have some degree of NAC necrosis and 2.0% of cases complicated by complete necrosis leading to NAC loss.  Sixteen studies [representing 2213 mastectomies] reported rates of skin flap necrosis, which occurred in 9.5% of cases.” |
| Endara 2013^16^ | **PubMed and Ovid databases, 1970 to 1 January 2013** | **48 studies, 5166 patents** | “Nipple-sparing mastectomy appears to be an oncologically safe option for properly selected patients, with low rates of locoregional and distant metastasis.  Overall complication and nipple necrosis rates are affected by incision location and reconstruction method.” |

**Table S2 Quality of previous systematic reviews according to AMSTAR**

| AMSTAR Criterion | Zhang et al 2015 | Piper et al 2013 | Endara et al 2013 |
| --- | --- | --- | --- |
| 1]. Was an ‘a priori’ design provided? | **NO** | **NO** | **NO** |
| 2]. Was there duplicate study selection and data extraction? | **NO (only data extraction)** | **NO** | **YES** |
| 3]. Was a comprehensive literature search performed? | **NO (single database only)** | **NO (only two databases)** | **NO (only two databases)** |
| 4]. Was the status of publication [i.e. grey literature] used as an inclusion criterion? | **NO** | **NO** | **NO** |
| 5]. Was a list of studies [included and excluded] provided? | **NO (included only)** | **NO  (included only)** | **NO** |
| 6]. Were the characteristics of the included studies provided? | **YES** | **YES** | **YES** |
| 7]. Was the scientific quality of the included studies assessed and documented? | **NO** | **NO** | **YES** |
| 8]. Was the scientific quality of the included studies used appropriately in formulating conclusions? | **NO** | **NO** | **YES** |
| 9] Were the methods used to combine the findings of studies appropriate? | **YES** | **N/A** | **YES** |
| 10]. Was the likelihood of publication bias assessed? | **YES** | **NO** | **NO** |
| 11]. Was the conflict of interest stated? | **YES** | **YES** | **YES** |
| TOTAL | 4 | 2 | 6 |

**Table S3 Electronic search strategy**

| **#** | **Searches** | **Results** |
| --- | --- | --- |
| 1 | exp Breast Neoplasms/su [Surgery] | 31262 |
| 2 | exp Mastectomy/ | 24336 |
| 3 | [breast* adj2 [surg* or reconstruct*]].ti,ab. | 15404 |
| 4 | mastectom*.ti,ab. | 16370 |
| 5 | 1 or 2 or 3 or 4 | 50022 |
| 6 | [[nipple* or areola* or nac] adj3 [spare? or sparing or preserv* or reposition*]].ti,ab. | 513 |
| 7 | 5 and 6 | 424 |
| 8 | Nipples/ and Organ Sparing Treatment/ | 21 |
| 9 | 7 or 8 | 426 |

**Table S4 Characteristics of the included studies**

| Study | Country | Patients  NSM/SSM | Mean or median age | Smoking status | Comorbidities |
| --- | --- | --- | --- | --- | --- |
| Wei 2016^30^ | USA | 52/202 | NSM – 45 (23-64)  SSM – 46 (23-72) | NSM - 30.8% SSM - 30.2% | NSM - 7.7% DM or HTN  SSM - 7.9% DM or HTN |
| Lemaine 2015^31^ | USA | 106 total  72/103 breasts | 48 (range 25-76) for whole population | NR | NR |
| Kim 2015^32^ | South Korea | 79/71  Unilateral patients only | 44 (26-65)  for whole population) | NR | NR |
| Poruk 2015^33^ | USA | 130/131 patients, 204/195 breasts | NSM – 45 +/- 12  SSM – 55 +/-15 | NSM - 20%  SSM - 24.4% | NSM - 5.4% diabetic  SSM - 13.7% diabetic |
| Yoo 2014^34^ | South Korea | 383/581 | NR | NR | NR |
| Gould 2013^35^ | USA | 73/NR  40 bilateral NSM so 113 NSM breasts (62 risk reduction) / 120 SSM breasts | NSM – 47 +/- 9  SSM – 50 +/-8 | NSM – Current/ prior 22 (19.5%)  SSM - NR | NSM – 12 (11%) had DM or HTN  SSM - NR |
| Burdge 2013^36^ | USA | 39/21 patients with locally advanced disease | NSM - 48 +/-10  SSM – 54 +/- 10 | 15% across both groups | NR |
| Moyer 2012^37^ | USA | 26/20 patients, 40 NSM breasts (3 patients had bilateral prophylactic NSM) | NSM – 42  SSM - 44 | NR | NR |
| Boneti 2011^38^ | USA | 152/41 patients, 281/227 breasts  215 bilateral 78 unilateral | NSM – 51 +/- 11  SSM – 53 +/-12 | 3.4% across both groups | NR |
| Jeon 2010^39^ | South Korea | 133/69 | NSM – 40  SSM - 40 | NR | NR |
| Kim 2010^40^ | South Korea | 152/368 | NSM – 42 +/- 7  SSM – 43 +/- 7 | NR | NR |
| Gerber 2009^43^ | Germany | 60/48 | NSM – 46 +/- 10  SSM – 48 +/- 10 | NR | NR |
| Ueda 2008^42^ | Japan | 33/41 | NSM – 44  SSM - 47 | NR | NR |
| Gerber 2003^41^ | Germany | 61/51 | NSM – 49 +/- 8  SSM – 50 +/- 9 | NR | NR |

(NR = not reported, HTN = hypertension, DM = Diabetes Mellitus)

**Table S5 Oncological profiles of patients included in the studies**

| Study | Mean tumour-nipple distance | Tumour distribution | Tumour Stage |
| --- | --- | --- | --- |
| Wei 2016^30^ | >1cm for both groups | NR | Early stage (0, I, II) NSM (100%, n = 40), SSM (96.8%, n = 182)  Late stage (III, IV) SSM (0%, n = 0), NSM (3.2%, n = 6) |
| Lemaine 2015^31^ | NR | NR | Stage 0 31 (17.7%)  Stage I 29 (16.6%)  Stage II 21 (12%)  Stage III 3 (1.7%)  Not applicable (risk reduction/ benign indication) 91 (52%) |
| Kim 2015^32^ | NR | NR | In situ carcinoma 16 (10.7%16)  Stage I 57 (38%)  Stage II 67 (44.7%)  Stage III 10 (6.7%) |
| Poruk 2015^33^ | NSM - 3.65cm +/- 1.61  SSM - 3.87 +/- 2.36 | NR | Stage 0 NSM (23.3%, n = 24) SSM (8.5%, n = 10)  Stage I NSM (35%, n = 36), SSM (28.8%, n = 34)  Stage II NSM (33%, n = 34), SSM (36.4%, n = 43)  Stage III NSM (6.8%, n = 7), SSM (22.9%, n = 27)  Stage IV NSM (1.9%, n= 2), SSM (3.4%, n = 4) |
| Yoo 2014^34^ | NR | NR | DCIS: NASSM (18.0%, n = 69), SSM (18.2%, n = 106)  Stage I: NASSM (36.0%, n = 138), SSM (37.0%, n = 215)  Stage IIa: NASSM (25.6%, n = 98), SSM (23.6%, n = 137),  Stage IIb: NASSM (12.3%, n = 47), SSM (11.0%, n = 64)  Stage >III: NASSM (8.1%, n = 31), SSM (10.2%, n = 59) |
| Gould 2013^35^ | NSM - Mean not provided but 16 patients <5cm and 30 patients ≥ 5cm  SSM - NR | NR | NSM 51 therapeutic:  DCIS 10 (8.8%)  Invasive disease ≤2cm: 22 (19.4%, n = 26) SSM NR  Invasive disease >2cm: 19 (16.8%))  SSM – NR |
| Burdge 2013^36^ | NR | NR | High risk, stage IIB or III  Pre-chemo size 4.6 +/-2.9 SSM  Pre-chemo size 3.4 +/- 2.2 NSM  47 neoadjuvant chemo/hormonal therapy  4/60 prior RT, 56/60 received PMRT |
| Moyer 2012^37^ | NR | NR | Stage 0 7 (29%)  Stage I 9 (38%)  Stage IIa 6 (25%)  Stage IIb 1 (4.2%)  Stage III 1 (4.2%) |
| Boneti 2011^38^ | NR | NR | 1.9cm +/-1.6 NSM  2.1cm +/- 1.7 SSM  45 (30.7%) of whole population were node positive |
| Jeon 2010^39^ | Nipple was sacrified, if tumor cell is within 2mm from nipple-areolar resection margin. | NR | T0 stage – 34 (25.6%) NSM vs. 22 (27.7%) SSM T1 stage – 67 (50.3%) NSM/ 34 (50.0%) SSM T2 stage – 32 (24.1%) NSM/ 13 (22.3%) SSM  N0 stage – 105 (78.9%) NSM/ 58 (84.1%) SSM N1 stage – 28 (21.2%) NSM/ 11 (15.9%) SSM |
| Kim 2010^40^ | NR | Unifocal – 344 (66.2%) patients  Multifocal – 176 (33.8%) patients | Stage 0 – 19 (12.5%) NSM/ 65 (17.7%) SSM  Stage I – 70 (46.1%) NSM/ 150 (40.8%) SSM   Stage II – 55 (36.2%) NSM/ 121 (32.9%) SSM  Stage III – 8 (5.3%) NSM/ 32 (8.7%) SSM |
| Gerber 2009^43^ | NSM - Tumour margins of greater than 2cm from the nipple  SSM - NR | NR | Stage 0 or 1 – 11(18%) NSM/ 11 (23%) Stage II – 44 (73%)/ 36 (75%) SSM  Stage III – 5 (8%) NSM/ 1 (2%) SSM |
| Ueda 2008^42^ | NR | NR | Tis stage – 5 (15%) NSM vs. 2 (5%) SSM T1 stage – 12 (36%) NSM/ 20 (49%) SSM T2 stage –15 (45%) NSM/ 18 (44%) SSM T3 stage – 1 (3%) NSM/ 1 (2%) SSM  N0 stage – 31 (94%) NSM/ 40 (98%) SSM N1 stage – 1 (3%) NSM/ 1 (2%) SSM N2 stage – 1 (1%) NSM/ 0 (0%) SSM |

**Table S6 Summary of complication rates in NSM and SSM groups (breasts)**

| Complication | NSM | SSM |
| --- | --- | --- |
| Partial or Complete Nipple Necrosis Rate | 15.0% (116/773) | N/A |
| Mastectomy Skin Flap necrosis | 3.4% (26/773) | 3.9% (28/724) |
| Haematoma | 0.5% (4/773) | 1.0% (7/724) |
| Infection | 1.4% (11/773) | 1.5% (11/724) |
| VTE | 0.4% (3/773) | 0.3% (2/724) |
| Blood transfusion | 0.3% (2/773) | 0.4% (3/724) |
| Seroma | 0% | 0.6% (4/724) |
| Not-specified/other | 0.8% (6/773) | 0.4% (3/724) |
| Overall complication rate | **23.4%** (181/773) | **13.5%** (98/724) |

**Table S7 Clinician and patient-reported outcomes**

| Study | NSM | SSM |
| --- | --- | --- |
| Wei et al  2016 | BREAST-Q mean scores: Psychological well-being 83.2(±20.8)* Sexual well-being 65.5(±23.6)* Physical well-being 80(±14.2) Satisfaction with breast  66.2(±18.5) Satisfaction with outcome 76.6(±20.5) | BREAST-Q mean scores:  Psychological well-being 75.5(±18.8)* Sexual well-being 57.4(±21.1)* Physical well-being 77.2(±14.1) Satisfaction with breast 65.6(±18.8) Satisfaction with outcome 76.4(±19.4)  *=statistically significant |
| Lemaine et al  2015 | Not reported | Not reported |
| Kim et al  2015 | Not reported | Not reported |
| Poruk et al  2015 | Not reported | Not reported |
| Yoo et al  2014 | Not reported | Not reported |
| Gould et al  2013 | Not reported | Not reported |
| Burdge et al  2013 | Women were asked to rate their cosmetic result on a visual analogue scale from 0 to 10 in comparison to their preoperative breast. Data were available for 8 of 39 (21%). The average rating was 8 out of 10 by the patient and 9 out of 10 by the physician. | Not reported |
| Moyer et al  2012 | Assessed by 3 surgeons and a medical student using subscales originally described by Garbay et al. SSM patients had completed nipple reconstruction.   \| Aesthetic Score \| NSM  N=20 \| SSM  N=20 \| \| --- \| --- \| --- \| \| Symmetry \| 1.32 \| 1.27 \| \| IMF \| 1.41 \| 1.24 \| \| Volume \| 1.53 \| 1.56 \| \| Contour \| 1.44 \| 1.24 \| \| Nipple \| 1.45* \| 1.11* \| \| Overall \| 7.13 \| 5.98 \|   *Nipple score significantly better for NSM | |
| Boneti et al  2011 | Women asked to rate their cosmetic result on a scale from 0 to 10 in comparison with their preoperative breast.  Data available for 45/152 (30%) Score = 9.2+/- 1.1.  4/45 (9%) reported their final cosmetic result above 10 (i.e. better than their original breasts). | Data available for 11/141(8%)  Score = 8.3+/- 1.9. |
| Jeon et al  2010 | Not reported | Not reported |
| Kim et al  2010 | Not reported | Not reported |
| Gerber et al  2009 | Tool - subscales according to Lowery et al – surgeons evaluated breasts and awarded up to 2 points for each category: volume, contour, placement of breast mound, and inframammary fold, making a potential maximum of 8 points. Results were defined as: 7-8 = excellent; 6-6.9 = good; 5-5.9 =fair; <5 = poor.   \|  \| NSM \| SSM \| \| --- \| --- \| --- \| \| Excellent \| 31/60 (52%) \| 23/48 (48%) \| \| Good \| 27/60 (45%) \| 19/48 (40%) \| \| Fair/poor \| 2/60 (3%) \| 6/48 (13%) \| | |
| Ueda et al  2008 | Tool - scale established by the Japanese Breast Cancer Society for assessment by clinicians. By assessment of photographs at a mean of 12 months post op. No statistically significant difference between NSM and SSM   \|  \| NSM \| SSM \| \| --- \| --- \| --- \| \| Excellent \| 6/28 (21%) \| 3/28 (11%) \| \| Good \| 15/28 (54%) \| 14/28 (50%) \| \| Fair \| 4/28 (14%) \| 9/28 (32%) \| \| Poor \| 3/28 (11%) \| 2/28 (7%) \|   Patient reported outcome using QOL-ACD and QOL-ACD-B scores   \|  \| NSM \| SSM \| \| --- \| --- \| --- \| \| Social activity \| 95 \| 92 \| \| Physical aspects \| 88 \| 83 \| \| General condition \| 79 \| 74 \| \| Bodily pain \| 76 \| 77 \| \| Body image \| 87 \| 85 \| \| Body image 2 \| 60 \| 53 \| \| Sexual aspects \| 60 \| 64 \| | |
| Gerber et al  2003 | Rated by surgeons:  Excellent 45/61(74%)  Good 16/61 (26%) | –  Excellent (30/51 =(59%)  Good 11/51 (22%)  Fair 10/51 (20%) |

**Fig. S1 Number of articles published per year and indexed by Scopus under the search term ‘nipple-sparing mastectomy’**
